# Effects of three home-based exercise programmes regarding falls, quality of life and exercise-adherence in older adults at risk of falling: protocol for a randomized controlled trial

**DOI:** 10.1186/s12877-018-1021-y

**Published:** 2019-01-14

**Authors:** Anne-Gabrielle Mittaz Hager, Nicolas Mathieu, Constanze Lenoble-Hoskovec, Jaap Swanenburg, Rob de Bie, Roger Hilfiker

**Affiliations:** 1 Caphri - Care and Public Health Research Institute and Department of Epidemiology Maastricht University, Leukerbad, Netherlands; 2 HES-SO Valais-Wallis, School of Health Sciences, Physiotherapy, Rathausstrasse 8, Leukerbad, VS Switzerland; 3 CUTR Sylvana, Chemin de Sylvana 10, Epalinges, Switzerland; 4 Interdisciplinary Spinal Research ISR, Department of Chiropractic Medicine, Balgrist University Hospital, Zürich, Switzerland

**Keywords:** Older adults, Home-based exercises programmes, Falls, Quality of life, Exercise-adherence

## Abstract

**Background:**

Fall prevention interventions with home-based exercise programmes are effective to reduce the number and the rate of falls, by reducing risk factors. They improve balance, strength, function, physical activity, but it is known that older adults’ exercise adherence declines over time. However, it is unclear which delivery-modalities of the home-based exercise programmes show the best adherence and the largest effect.

We created a new home-based exercise programme, the Test-and-Exercise (T&E) programme, based on the concepts of self-efficacy and empowerment. Patients learn to build their own exercise programme with a mobile application, a brochure and cards, as well as with eight coaching sessions by physiotherapists.

The main objective of this study is to compare the T&E programme with the Otago Exercise Programme and the recommendation-booklet and exercise-cards of Helsana regarding incidence of falls. Other outcomes are severity of falls, functional capacities, quality of life and exercise-adherence.

**Methods:**

The design of this study is a Swiss multicentre assessor blind randomized controlled trial. A block-randomization, stratified in groups for age and risk of fall categories, will be used to allocate the participants to three groups. The targeted study sample consists of 405 older adults, ≥ 65 years of age, living in the community and evaluated as at “risk of falling”. Experimental group will receive the T&E programme (*N* = 162). Second group will receive the Otago programme (*N* = 162) and the third group will receive the Helsana programme (*N* = 81). All interventions last six months. Blinded assessors will assess participants three times: at baseline before the start of the intervention, after six months of intervention and a final assessment after twelve months (six months of follow up).

**Discussion:**

Although home-based exercises programmes show positive effects in fall prevention in elderly persons, existing programmes do often not include patients in the decision-making process about exercise selection. In our programme the physiotherapist and the older adult work together to select the exercises; this collaboration helps to increase health literacy, pleasure of exercising, and empowers patients to be more autonomy.

**Trial registration:**

ClinicalTrials.gov: NCT02926105, First Posted: October 6, 2016, Last Update: November 11, 2016: Enrolment of the first participant.

## Background

Falls are one of the major health problems affecting the quality of life in older adults [1, 2] and are particularly common and burdensome in the elderly population [3]. Of community-dwelling older adults over 64 years of age, 28–35% fall each year and the frequency of falls increases with age and frailty level [4], up to 50% in the elderly aged more than 80 years [5]. It represents a serious public health problem due to high healthcare demands and related healthcare costs and has a major impact on the individual patient [6].

Consequences of these falls are somatic, psychological, social and economic [7, 8]. They induce loss of confidence and fear of further falls [9], chronic pain, loss of independence and reduced quality of life [10] as well as economic cost greater than policy makers appreciate [11]. In 10 to 20% of the cases, falls result in bone fractures and head injuries [12] and can lead to increased mortality [7, 12]. It may not be possible to prevent falls completely, but people who tend to fall frequently may be enabled to fall less often [13].

However, for the older adults, living in their own home or in institution, physical and psychological impairment and functional limitations worsen significantly the quality of life [14]. Thus, in older adults at risk to fall, balance, functional mobility, muscle strength and fear of falling are associated with quality of life [15–21].

Regular physical activity could be a way to slow down the decline and maintain or even increase personal autonomy and quality of life [22]. According to the recommendations and strategies for promoting exercise in older adults [23], elderly people at risk could also profit from adapted exercise programmes [24–26]. Fall prevention interventions that include exercise programmes are recognized as effective to reduce the incidence of falls in elderly community-dwelling individuals [13, 27–29], seem to prevent injuries caused by falls [30] and show significant improvement in quality of life in frail older adults [31], as well as in women with osteopenia or osteoporosis [32]. However, the question about optimal methods for exercise delivery, exercise selection and exercise progression is not yet conclusively answered.

Home-based exercise programmes appear to reduce rate of falls [33], number of fallers, risk factors for falls, and risk of death [34] by improving balance, leg strength, function, physical activity [35] and balance confidence in older adults living in the community [36].

To maintain the effects of the home-based exercise programmes, older people should continue as long as possible with these exercises. However, older adult’s adherence to exercise interventions declines over time [37, 38]. A variety of factors determine, increase or decrease adherence to exercise in older adults [39]. These factors could be psychological and cognitive, behavioural, physical or simply organisational. The strongest motivator affecting exercise adherence in older adults is *self-efficacy* [39] and the integration of the *self-efficacy* concept in exercise programmes to enable individuals to continue with the programme is highly recommended [37, 40].

Because *self-efficacy* is a modifiable factor, project promoters and researchers could enhance the adherence in falls-prevention interventions using specific recommendations [41]. There also is a range of factors affecting the implementation of fall-prevention practices. They are related to older adults, families, healthcare professionals and healthcare systems [42]. General considerations consist in raising awareness in the population and in health professionals [7, 43], promotion of benefits of exercises in older people [43, 44] and consideration of the individual lifestyle, values and capabilities [7, 43, 44]. More specific, encouraging confidence in self-management by giving older adults an active role [43] and providing moderate follow-up visits [7, 45] could improve adherence to home exercise programmes for prevention of falls. Currently, with the advent of e-technology, several studies use mobile applications [46–49] to improve adherence and motivate older adults living in their own home to exercise. Therefore, we developed a new home-based exercise programme, based on the concepts of self-efficacy and of empowerment, delivered on a mobile application. Because older people have to test the exercises before starting to train, this programme is named Test and Exercise (T&E).

The aim of this study is to compare the effects of the T&E exercise programme with the effects of the Otago and Helsana exercise programmes in older adults at-risk for falling, while living in their own home.

Our hypothesis is that the reference intervention (Otago) is superior to the usual care (Helsana) and that the experimental intervention (T&E) is non-inferior to the reference intervention.

## Methods/design

### Study design

The study design is a multicentre randomized controlled trial, assessor blinded, with three groups. It follows the recommendations of SPIRIT 2013 [50]. The trial is executed at the homes of the participants in four Swiss regions: Zurich, Vaud, Bas-Valais and Haut-Valais.

### Participants

The population consists of older adults > 65 years, living in their own home and identified as at-risk of falling. Table 1 shows the inclusion and exclusion criteria.

**Table 1:** Inclusion and exclusion criteria

Inclusion criteria	Exclusion criteria
Older adults aged ≥ 65 years living independently at home	Severe vision impairments that do not permit the reading of the exercise-programme booklet and that do not permit the completing of the monthly diaries
Able to walk without mobility aids in their home	In physiotherapy treatment that trains balance
History of fall in the previous 12 months or perceiving fear of falling (≥ 20 points [98] on FES-I [63])	Cognitive impairment (< 24 points [99] on the Minimal Mental State Examination [100])
Good understanding of the Trial language (French or German)	Contraindication given by the referring physician

### Recruitment, randomization, blinding and treatment allocation

Caregivers (medical doctors, nurses, physiotherapists) in partner hospitals, health-care centres and medical offices will recruit the participants. To achieve adequate enrolment to reach target sample size, we projected to enrol a quarter of the sample size in each region (101/region) for three years (from August 2016 to September 2019). In addition, the local coordinators will meet the recruitment partners twice a year. They will give a feed-back about the progress state on the trial and motivate them to continue the recruitment.

The local coordinators will verify the eligibility criteria (Table 1) at the home of the participants.

The final decision on inclusion or exclusion and randomization takes place after the baseline assessment. A researcher who is not involved in the study creates an allocation list with a computer generated random sequence using the command “ralloc” within Stata (StataCorp, college Station, Texas, USA), version 14.2. A block-randomization is executed stratified in groups of age (65–79; > 80 years old) and in risk of fall categories [51] (low-moderate; high). If a couple living in the same house will participate, both persons will be randomized in the same intervention group [52].

The randomization was integrated in the software for Research Electronic Data Capture (REDCap [53]) and is concealed to all members of the research team. The project assistant manually triggers the randomization after the baseline assessment. The local coordinators will take note of the allocation group in REDCap.

### Informed consent

All eligible patients are informed about this study and given the time they need to consider participation. The investigators and the local coordinators may be approached for questions. Patients who accept to participate will sign an informed consent form and transmit it to the local coordinator.

### Interventions

Special trained physiotherapists will take care of the patients. All the involved physiotherapists in this study are used to treat older adults at their own home.

### Experimental intervention

The experimental intervention consists of a home-based exercise programme Test-and-Exercise (T&E) delivered by specially trained physiotherapists. The development of this programme was carried out in several successive phases. We first established a list of 48 physical exercises that train balance control, mobility and strength of the body as well as functional exercises. We then asked 63 older adults to perform these exercises and to evaluate the perceived difficulty of each exercise using items with five response options: (0 = “no difficulty”; 1 = “some difficulty”; 2 = “difficult”; 3 = “very difficult”; 4 = “too difficult, I don’t do it”) [54]. At the same time, observer physiotherapists evaluated the quality of the task (0 = “perfectly performed”; 1 = “good performed with light deviation”; 2 = “performed with moderate deviation”; 3 = “badly performed”; 4 = “not performed”). This reliability study showed sufficient reliability of both items to be used in older persons to monitor exercise difficulty regarding the concepts of *self-efficacy*, *self-confidence* and *empowerment* to increase the motivation and the self-confidence [55].

We then developed a first Android application containing videos of the physical exercises of the T&E programme in addition to the T&E exercise booklet. We integrated the self-perceived difficulty items so that the participants have to rate the difficulty of the task before they include them in their own home-based exercise programme.

After this, we conducted a feasibility-study in 2014–2015 [56] where 19 participants were randomized in two groups: Intervention group T&E and Control group Otago. The results of this feasibility-study allowed us to prepare this randomized controlled study.

**Fig. 1:**
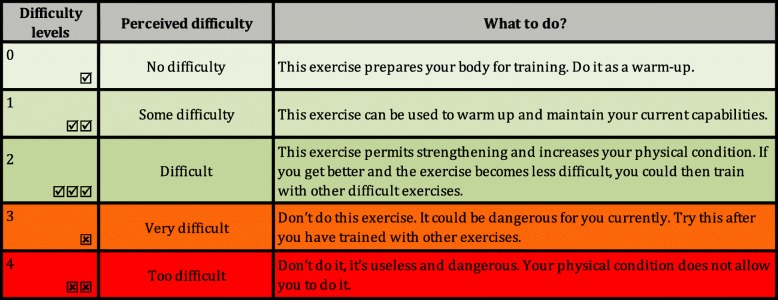
Recommendations for create your home-based exercise-programme

The T&E programme is delivered in a booklet with pictures and task descriptions, and on a digital tablet. The current programme contains 50 physical tasks grouped under fourteen topics related to home objects or activities. Each topic contains three or four tasks, ranged according to increasing difficulty. Unlike usual home-based exercise programmes, the physiotherapists do not prescribe exercises: they help and coach the participants to build their own exercise programme while ensuring safety and security (see Table 2). The patient chooses the tasks he/she wants to do, does it once as “test” and evaluates the perceived difficulty. The rating of the perceived difficulty classifies the task in two groups of exercises: those that can be included in their programme and those that should not be included. The Fig. 1 shows how to use the perceived difficulty to build the exercise-programme.

**Table 2:** The innovative elements of the T&E programme

The innovative elements of the T&E programme	
1. Empowerment/Self-efficacy	Patient express his needs and fixes his goals for his/her own exercise programme
2. Learning autonomy step-by-step	Physiotherapists coach patients to learn how to manage his/her exercise programme
3. Test before Exercise	The evaluation of the perceived difficult allows the construction of the exercises programme in security to be sufficiently challenging without being dangerous
4. No prescription of exercises	Based on the perceived difficulty of the exercises, the patients decide which exercises they include in their own programme
5. Retest regularly the tasks	The re-evaluation of the perceived difficulty allows to see the progression of the capabilities
6. Use of an Android application on digital tablet	The digital tablet permit the visualization of the tasks at any moment, as Test and as Exercise
7. Fifty physical tasks	llows choices and adaptation in long-term

Physiotherapy treatment is focused on increasing motivation and exercise adherence, working on the self-efficacy, the self-confidence and the autonomy of the patients. The implementation of the T&E programme is planned with eight physiotherapy sessions and four phone calls in six months. At each session the physiotherapists revise the exercises execution, verify that the diaries are correctly completed and repeat the recommendations for the training. At each phone call, the physiotherapists ask about the exercises, if the patient encounter difficulties and encourage them to train themselves.

The physiotherapists for the implementation of the T&E programme are trained in a basic training of half a day. The training focuses on i) Encouragement of the autonomy of the participant, ii) The significance of the evaluation of the perceived difficulty, iii) The work as coach for the participant, iv) Stimulation for exercises adherence: not too much at the same time, but regularly, v) Safety of the environment where the patient will train, vi). All physiotherapists will participate in a yearly refresher course and experience sharing session.

### Control interventions

To evaluate the T&E programme, we will compare it with two established interventions.

The first intervention consists of the implementation of the Otago exercise programme (OEP) [57]. This programme was developed in New-Zeeland in the 2000’s to prevent falls in older adults. It contains 22 exercises: five for warming-up, five for muscle strengthening of the lower limbs and twelve for balance training. In addition, it recommends walking sessions between the training days. OEP shows significant results in reducing the risk of death and falling in older community-dwelling adults but moderate exercise adherence [34].

The physiotherapists that implement the Otago programme are trained in a half-day basic training. The training consists of information about the Otago programme and how to deliver it to the patients with the original recommendations of Prof Campbell and Robertson [57]. In this training emphasis is put on prescription and control of the exercises. The physiotherapists will adapt the programme at every home session. The implementation of the Otago programme is similarly planned as the T&E program: eight home session and four phone calls in six months. At each session the physiotherapists revise the exercises execution, verify that the diaries are correctly completed and repeat the recommendations for the training. At each phone call, the physiotherapists ask about the exercises, if the patient encounter difficulties and encourage them to train themselves.

The second intervention (“Helsana”) is considered as usual care. It consists of a booklet with safety advice and twelve exercise-cards for “Going safely”, as constructed by the Swiss health care insurance Helsana. Helsana agreed to the use of their programme in this study (no financial support was provided). It contains five exercises in sitting position; six exercises in standing position and one stand up exercise. This programme is self-directed. It has never been evaluated in a trial. Physiotherapists will demonstrate the programme during one home session while they explain the recommendations and they will do all the exercises with the patient to ensure the security. They will then call the patients four times in a six-week interval, to ask if they encounter problems with the exercises.

In cases where the practice of home-based exercises could potentially harm the patients, the local coordinator could exclude the patients of the trial.

The innovative elements of the T&E programme are set out in Table 2 and these are the main distinguishing factors between the experimental intervention and both control interventions.

Even though participation of the patient in his/her treatment is considered as important to improve self-efficacy and exercise adherence, the physiotherapists often tend to apply a paternalistic approach rather than involving the patient [58]. The T&E programme offers the opportunity to the physiotherapists to experiment with a new way to work with patients.

### Outcomes

The primary outcome “falls incidence” and exercise frequency will be assessed prospectively and for twelve months with self-recorded monthly diaries. If a patient decides to stop his participation (completing monthly diaries and exercise training), the physiotherapist will ask him to participate at the intermediary or/and finale assessment. Physiotherapist-assessors who are unaware of group allocation (“blinded assessors”) will assess the secondary outcomes at baseline, at the six-month intermediary assessment, and at the twelve-month final assessment. All assessors are independent physiotherapists and specially trained to perform the assessments.

To ensure that the assessors remain unaware of the group allocation, patients are told not to talk about their treatment with the assessor.

The main outcome is the incidence of falls for twelve months. For this study we define a fall as “*an unexpected event in which the participant comes to rest on the ground, floor, or lower level, with or without injury*” as recommended in the systematic review of Hauer et al. [59]. The history of falls (number, circumstance (Where? *Inside OR outside;* When? *Day OR night;* How? *Describe shortly the event*) and severity) in the previous twelve months will be assessed at baseline. During the study, falls will be prospectively self-reported (number, date, circumstance and severity) in monthly diaries [60]. To ensure that the patients complete the diaries, the physiotherapists will check them at each visit and ask about the diaries during each phone call [59]. In addition, falls events (number, circumstance and severity) will be asked during the intermediary assessment and at the final assessment. The diaries will be sent to the coordinators. The coordinators will send them in PDF version to the project-assistant. She will enter the data in REDCap software once a month.

Secondary outcomes include severity of falls, fear of falling, gait, strength and balance and quality of life. They will be assessed at baseline, at the intermediary assessment and at the final assessment. They also include exercise-adherence. This outcome will be assessed at twelve months.

The severity of falls will be self-reported in the monthly fall diaries using the four categories defined by Schwenk et al. [61]: a. Serious injury (requiring accident and emergency or inpatient treatment); b. Moderate injury (requiring a medical/health professional examination); *C. minor* injury (reduction in physical function for at least three days); d. No injury.

Fear of falling will be measured on a visual analogic scale (0 = no fear at all to 10 = extreme fear) [19, 62] and on the Falls Efficacy Scale-International FES-I [63]. FES-I measures the level of concern about falling during social and physical activities of daily living, inside and outside the home whether or not the person actually does the activity. This 16-item questionnaire offers a response format consisting of 4 levels ranging from “*not at all concerned*” to “*very concerned*”. It is a valid and reliable tool showing a good sensitivity to detect changes in concerns about falling [64]. The score ranges from 16 to 64: the higher the score, the more concerned one is about falling.

Gait, strength and balance will be assessed with five tests of which the three first are recommended as part of the STEADI initiative of the Centers for Disease Control and Prevention (CDC) [65]:

1. The “Timed Up and Go” test (TUG) [66–68] assesses lower extremity function, mobility and fall risk. It is not only associated with motor performance but also with cognitive function [69]. It is associated with history of past falls, but its predictive ability for future falls is limited [70] so it may be used in combination with other tests in fall risk assessment [71]. Patients sit on a chair and on the command “Go”, they have to stand up, walk three meters at a comfortable and sage pace, turn, walk back to the chair and sit down. The time is measured in seconds, begins at “Go” and ends when the patient is seated. The patients will perform the task twice. The average of both scores will be used in the analysis.
2. The “Four-Test balance scale” [72], also called “Four Stage Balance Test” (FSBT) by the CDC, consist of standing in four progressively more difficult stances: 1. Feet side-by-side; 2. Partial tandem; 3. Tandem and 4. On one foot. If the patient can hold a position for ten seconds without moving his/her feet or needing support, he/she goes on the next position. If not, the test is stopped. The level reached will be noted.
3. The “Five Time Sit to Stand” test (FTSTS) was described to test the ability to rise from a chair in a short battery of physical-performance tests [73]. It is a valid measure of dynamic balance and functional mobility in older adults and has excellent relative and absolute reliability and reproducibility [74]. With a cut-off value at 12 s it demonstrates a reasonable sensitivity and specificity in identification of multiple fallers [75]. Patients sit on a straight-backed chair placed next to a wall; they will be asked to fold their arms across their chest and to stand up five times in a row as quickly as possible, when the assessor says “Go”. Patients have to come to a full standing position each time they stand up and have to sit down without touching the back of the chair. The time is measured in seconds, begins at “Go” and ends with the fifth seat. The patients will perform the task twice. The average of both scores will be used in the analysis.
4. The “Functional Reach” test (FR) [76–78] was designed to identify frail older people and elders at risk of falling. It measures the maximal distance that a person can reach forward while standing. The patient will stand next to a wall with the ipsilatéral arm at 90° flexion with fingers extended. The assessor will mark a point where the third finger is at the initial position on a static squared paper against the wall. The command is “*Lean forwards as fare you can without taking a step, bending the knee or raising the heels to bring your hand forward as far as possible*”. At the maximal anterior leaning position, the assessor marks a second point. The horizontal distance between the two points will be measured in centimetres. The patients will perform the task three times. The average of the three scores will be used in the analysis.
5. The “Six-meter-walk” test (SMWT) will be used to assess gait speed. Patients will walk “*as fast as possible without running*” ten meters including 2-m approach, 6-m for the time measure and 2-m beyond the measure to ensure a constant walking speed across six meters [79–81]. The time will be measured in seconds and divided by 6 to calculate the gait speed in m/s. The patients will perform the task twice. The average of both measures will be used in the analysis.

Quality of life will be measured with the French and German version of the “Older People’s Quality of Life Questionnaire” OPQOL-35 [82]. The questionnaire consists of 35 items measuring quality of life based related to eight domains: 1: Life overall (4Q), 2: Health (4Q), 3. Social relationships (8Q), 4: independence, control over life, freedom (5Q), 5: home and neighbourhood (4Q), 6: Psychological and emotional well-being (4Q), 7: Financial circumstances (4Q) and 8: Culture and religion (2Q). The score ranges from 35 to 175: as higher the score, as better the quality of life.

Adherence to exercise will be prospectively assessed in monthly training diaries during 12 months where date, duration, type of exercises (including walking) and intensity of effort are noted. To ensure that the patients complete the diaries, the physiotherapists will check them at each visit and ask about the diaries during each phone call.

Confounding factors, like life situation, living place, physical activity habits and comorbidity will be documented at each assessment session.

### Patient flow

**Fig. 2:**
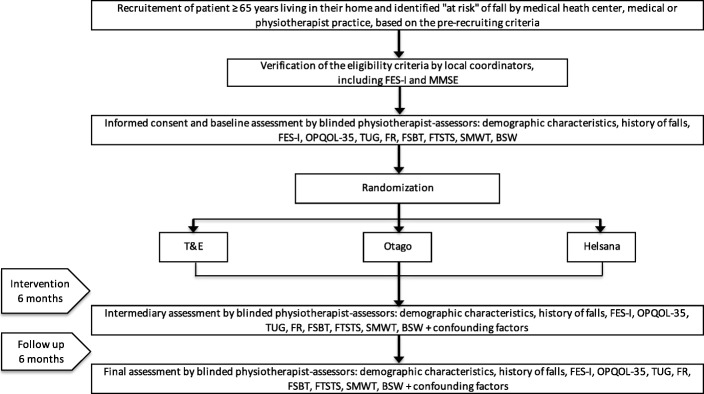
Patient Flow. FES-I: Falls Efficacy Scale - International; MMSE: Mini Mental State Examination; OPQOL-3: Older People’s Quality of Life Questionnaire; TUG: Timed Up and Go test; FR: Functional Reach Test; FSBT: Four Stage Balance Test; FTSTS: Five Times Site To Stand test; SMWT: Six Meters Walk Test; BSW: Base of Support Width

Figure 2 describes the patient flow. This trial will last 36 months, and individual patient participation is planned for twelve months. Patients will be recruited from autumn 2016 until autumn 2019. We expect to enrol 20–21 subjects in each of the four regions every six months to reach the sample size. The last follow-up phase should end in autumn 2020.

### Data management

All data will be directly entered in REDCap software. This software allows the protection of the confidentiality of the data during the study. As soon a patient gives his written consent he will receive an Identity Code (ID). The project-assistant will verify all data entries before validation. If data are missed, she will ask for complement. For data cleaning, statisticians will be blinded to group allocation. At the end of the study, all the data will be anonymised. The final trial dataset will be accessible to the Data monitoring committee for the analysis. 

### Sample size

To achieve our study objectives, the sample size calculation estimates 405 participants. With a significance level set at 5% (i.e. overall type-I error probability) and power of 80%, an assumed rate of falls of 0.5 for the T&E program, 0.79 for the OTAGO and 0.92 for the usual care group, a non-inferiority margin of 5% and an assumed dropout rate of 15%, we would need to include 162 participants in the groups A and B each, and 81 participants in the group C. We used the R package “ThreeArmedTrials” to calculate the sample size [83].

The allocation scheme of 2:2:1 is based on ethical and statistical considerations, following the recommendations of Mütze et al. [84] and Mielke et al. [85].

### Statistical analysis

Descriptive statistics will be used to present group characteristics on age, sex, body mass index, base of support width (BSW) [86, 87], and history of falls, activity of daily live, physical activity habits, and risk factors for falls.

According to statistical criteria of termination of the trial, we have no predefined stopping rules because we are not expecting risks that are different in the groups (i.e. no adverse event stopping criteria).

We will use an intention-to-treat analysis based on all randomized patients, with missing values and dropouts replaced by multiple imputation methods, as recommended by current guidelines on randomized controlled trials [88].

The primary analysis will be done to test for the incidence of falls. We follow the proposition of Röhmel and Pigeot [89], who showed that superiority of the experimental group (“T&E” in our case) over the reference treatment (“Otago” in our case) can be tested after successfully demonstrating non-inferiority (if stated a-priori in the protocol). To simultaneously demonstrate superiority of E (T&E) to R (Otago) and R (Otago) to P(Helsana) as well as the non-inferiority of E (T&E) to R (Otago), we will use negative binomial regression [90, 91] and follow an approach proposed by Mütze et al. [84]. A sensitivity analysis without multiple imputations of missing values will be performed.

For the primary analyses, we will adjust for the participants’ length of observation, center, age and risk for falls category [92]. We will perform a per protocol sensitivity analysis including time varying co-variables to reduce confounding and selection bias, e.g. due to differential censoring.

Further analyses will be performed to test the secondary endpoints quality of life (Overall score of OPQOL-35), severity of falls (Schwenk Scale), fear of falling (FES-I), functional capacities (TUG, FSBT, FTSTS, FR and SMWT), as well as adherence to the exercise recommendations (duration in min/week, frequencies/week).

We will perform a subgroup analysis based on age groups to test the secondary hypothesis that compliance in older group is better with the Otago compared to T&E and that in younger participants’ compliance is better in T&E compared to Otago.

The data monitoring committee will do interim analyses only for data quality evaluation and for monitoring of adverse events, i.e. no interim for effectiveness will be performed. The results of this analyses will be accessible for the steering committee.

The proportion of adverse events (other than falls) will be compared between the groups by an external and independent statistician.

All deviations from the statistical plan will be documented and an amendment will be sent to the ethical commissions as well as to Protocol Registration System ClinicalTrials.gov. The deviations from plan to actual analysis will also be reported in the final publication(s).

We will use multiple imputation methods using 50 imputed datasets under the assumption that missing data are missing at random. We will run sensitivity analyses to test the robustness of our replacement models and the robustness of the assumption “missing at random”.

### Adverse events

Because the participants of this study are older adults at risk of falling, they can experience falls or other health diseases due to the old age. If the patient, members of his family, any caregiver or the physiotherapist notes an AE like:

- Falls that require medical attention.

- Exacerbation of a pre-existing illness.

- Increase in frequency or intensity of a pre-existing episodic event or condition.

- Condition detected or diagnosed after the intervention, even though it may have been present prior to the start of the study.

- Continuous persistent disease or symptoms present at baseline that worsen following the start of the study.

they will inform the local investigator as soon as possible. The local investigator will collect the AEs with the complete description of the event on the formulary “Database for AI/AE/SAE”.

### Audits and inspections

The source data/documents will be accessible to auditors/inspectors from CEC in the HES-SO Valais-Wallis research offices in Leukerbad, Rathausstrasse 8. The principal investigator will answer the questions during the inspections. All involved parties will keep the participant data strictly confidential.

## Discussion

### Strengths

Because of the growing older population, physiotherapists will be more and more requested for intermediate care services including home-based models [93]. In this multicentre study we built a network with engaged health providers in vulnerable community dwelling older people. In this cross-disciplinary model, physiotherapists taking care of patients at their own home will bring their competences to improve function, independence and quality of life in this population.

In addition, this RCT evaluates the effects of three structured home-based exercise programmes of which two are implemented by physiotherapists. We expect larger reduction on the incidence of falls in these two programmes, indicating the efficient role of the physiotherapists with the patients. Because the T&E programme is person-centred and based on self-efficacy and empowerment, we expect a larger effect of T&E on quality of life and on exercise-adherence.

### Weaknesses

Even though coaching belongs to the most recommended intervention strategy to enhance patient participation [94] it is necessary to move the physiotherapy profession towards a greater degree of understanding and application of the principles of person-centred practice [95]. This is one of the aspects of the T&E home-based programme. It encourages the physiotherapists to see patients as partners in the process of treatment as well encouraging patients to take part in their process of treatment (e.g. selecting the tasks they want to train) need a change of the culture care [96]. Implementing a new treatment strategy is a challenging process. Physiotherapists will not automatically adopt a new strategy even after they have been trained [97]. In the context of the study yearly meetings and refresh courses are organized. The physiotherapists will have the opportunity to share their experiences under the supervision of members of the steering committee.

### Publication and dissemination policy

During the study, we will send a Newsletter twice a year to the participants and the field partners (recruitment institutions and physiotherapists).

As soon results are available, they will be communicated to the participants, to the field partners, to the technology and health care professionals. We also will communicate to the public health and social politicians, as well as to health insurances to promote this kind of interventions for preventing falls and injuries for the older people.

We project to participate at professional meetings and congress and to publish this protocol and the results of the trial in medical revues, especially revues for geriatrics, rehabilitation, and public health.

### Trial status

Enrolment into the study started on November 2016. We expect to complete the recruitment by the end of November 2019.

## Abbreviations

BSW: Base of support width
CDC: Centers for Disease Control and Prevention, National Center for Injury Prevention and Control, Division of Unintentional Injury Prevention
FES-I: Falls efficacy scale – international
FR: Functional reach test
FSBT: Four stage balance test
FTSTS: Five times sit to stand
MMSE: Mini-Mental State Examination
OEP: Otago exercise programme
OPQOL-35: Older People’s Quality of Life Questionnaire
REDCap: Research Electronic Data Capture
SMWT: Six Meter Walk Test
T&E: Test-and-Exercise home-based programme
TUG: Timed Up and Go test
